# Supplementary material for: Investigating the Key Trends in Applying Artificial Intelligence to Health Technologies: A Scoping Review
Source: PLoS One. 2025 May 15;20(5):e0322197. doi: 10.1371/journal.pone.0322197 (PMC12080793; doi:10.1371/journal.pone.0322197)
Supplement: Attachment — Submitted filename: PONE-D-24-54151_reviewer.pdf [file pone.0322197.s001.pdf]

PLOS ONE

Investigating the Key Trends in Applying Artificial Intelligence to Health Technologies: A Scoping Review

--Manuscript Draft--

**Manuscript Number:**	PONE-D-24-54151
**Article Type:**	Research Article
**Full Title:**	Investigating the Key Trends in Applying Artificial Intelligence to Health Technologies: A Scoping Review
**Short Title:**	Key Trends in AI Applications in Healthcare: A Scoping Review
**Corresponding Author:**	samah Tawil<br>Lebanese American University<br>Beirut, LEBANON
**Keywords:**	artificial intelligence, healthcare, benefits, methodologies, challenges
**Abstract:**	Background: The use of Artificial Intelligence (AI) is exponentially rising in the healthcare sector. This change influences various domains of early identification, diagnosis, and treatment of diseases. Purpose: This study presents a scoping review of different studies on the application of AI in the healthcare sector.<br>Methods: The review included 68 academic studies from different databases. After careful review and data analysis, AI methodologies, benefits and challenges, were summarized.<br>Results: Our results have shown that AI tools such as deep learning methods and machine learning continue to significantly improve accuracy and timely execution of medical processes. Benefits were discussed from both the organizational and the patient perspective in the categories of diagnosis, treatment, consultation and health monitoring of diseases. However, some challenges may exist, despite these benefits, and are related to data integration and patient safety.<br>Conclusion: The article examines the present status of AI in medical applications and explores its potential future applications. The findings of this review are useful for healthcare professionals to acquire deeper knowledge on the use of medical AI from design to implementation stage. However, a thorough assessment is essential to gather more insights into whether AI benefits outweigh its risks. Additionally, ethical and privacy issues need careful consideration.
**Order of Authors:**	samah Tawil
	Samar Merhi
**Additional Information:**	
**Question**	**Response**
**Financial Disclosure**<br><br>Enter a financial disclosure statement that describes the sources of funding for the work included in this submission. Review the submission guidelines for detailed requirements. View published research articles from *PLOS ONE* for specific examples.<br><br>This statement is required for submission and **will appear in the published article** if the submission is accepted. Please make sure it is accurate.	The author(s) received no specific funding for this work.

Powered by Editorial Manager® and ProduXion Manager® from Aries Systems Corporation

**Funded studies**<br>Enter a statement with the following details:<br>• Initials of the authors who received each award<br>• Grant numbers awarded to each author<br>• The full name of each funder<br>• URL of each funder website<br>• Did the sponsors or funders play any role in the study design, data collection and analysis, decision to publish, or preparation of the manuscript?<br><br>Did you receive funding for this work?	
**Competing Interests**<br><br>Use the instructions below to enter a competing interest statement for this submission. On behalf of all authors, disclose any competing interests that could be perceived to bias this work—acknowledging all financial support and any other relevant financial or non-financial competing interests.<br><br>This statement is required for submission and **will appear in the published article** if the submission is accepted. Please make sure it is accurate and that any funding sources listed in your Funding Information later in the submission form are also declared in your Financial Disclosure statement.<br><br>View published research articles from *PLOS ONE* for specific examples.	The authors have declared that no competing interests exist.

**NO authors have competing interests**<br><br>Enter: *The authors have declared that no competing interests exist.*<br><br>**Authors with competing interests**<br><br>Enter competing interest details beginning with this statement:<br><br>*I have read the journal's policy and the authors of this manuscript have the following competing interests: [insert competing interests here]*<br><br>* typeset	
**Ethics Statement**<br><br>Enter an ethics statement for this submission. This statement is required if the study involved:<br><br>• Human participants<br>• Human specimens or tissue<br>• Vertebrate animals or cephalopods<br>• Vertebrate embryos or tissues<br>• Field research<br><br>Write "N/A" if the submission does not require an ethics statement.<br><br>General guidance is provided below. Consult the submission guidelines for detailed instructions. **Make sure that all information entered here is included in the Methods section of the manuscript.**	The Ethics committee has waived the ethics approval

Powered by Editorial Manager® and ProduXion Manager® from Aries Systems Corporation

**Format for specific study types**<br><br>**Human Subject Research (involving human participants and/or tissue)**<br>• Give the name of the institutional review board or ethics committee that approved the study<br>• Include the approval number and/or a statement indicating approval of this research<br>• Indicate the form of consent obtained (written/oral) or the reason that consent was not obtained (e.g. the data were analyzed anonymously)<br><br>**Animal Research (involving vertebrate animals, embryos or tissues)**<br>• Provide the name of the Institutional Animal Care and Use Committee (IACUC) or other relevant ethics board that reviewed the study protocol, and indicate whether they approved this research or granted a formal waiver of ethical approval<br>• Include an approval number if one was obtained<br>• If the study involved *non-human primates*, add *additional details* about animal welfare and steps taken to ameliorate suffering<br>• If anesthesia, euthanasia, or any kind of animal sacrifice is part of the study, include briefly which substances and/or methods were applied<br><br>**Field Research**<br><br>Include the following details if this study involves the collection of plant, animal, or other materials from a natural setting:<br>• Field permit number<br>• Name of the institution or relevant body that granted permission	
**Data Availability**<br><br>Authors are required to make all data underlying the findings described fully available, without restriction, and from the time of publication. PLOS allows rare exceptions to address legal and ethical concerns. See the PLOS Data Policy and FAQ for detailed information.	Yes - all data are fully available without restriction

A Data Availability Statement describing where the data can be found is required at submission. Your answers to this question constitute the Data Availability Statement and **will be published in the article**, if accepted.<br><br>**Important:** Stating 'data available on request from the author' is not sufficient. If your data are only available upon request, select 'No' for the first question and explain your exceptional situation in the text box.<br><br>Do the authors confirm that all data underlying the findings described in their manuscript are fully available without restriction?	
**Describe where the data may be found in full sentences. If you are copying our sample text, replace any instances of XXX with the appropriate details.**<br><br>• If the data are **held or will be held in a public repository**, include URLs, accession numbers or DOIs. If this information will only be available after acceptance, indicate this by ticking the box below. For example: *All XXX files are available from the XXX database (accession number(s) XXX, XXX.)*.<br>• If the data are all contained **within the manuscript and/or Supporting Information files**, enter the following: *All relevant data are within the manuscript and its Supporting Information files.*<br>• If neither of these applies but you are able to provide **details of access elsewhere**, with or without limitations, please do so. For example:<br><br>*Data cannot be shared publicly because of [XXX]. Data are available from the XXX Institutional Data Access / Ethics Committee (contact via XXX) for researchers who meet the criteria for access to confidential data.*<br><br>*The data underlying the results presented in the study are available from (include the name of the third party*	All relevant data are within the manuscript and its Supporting Information files.

and contact information or URL).<br>• This text is appropriate if the data are owned by a third party and authors do not have permission to share the data.<br><br>* typeset	
Additional data availability information:	

Manuscript

Click here to access/download;Manuscript;manuscript - AI - Final version.docx

Investigating the Key Trends in Applying Artificial Intelligence to Health Technologies: A Scoping Review

TAWIL Samah, PHD[1,2]*; MERHI Samar, PHD[3]

1. Gilbert and Rose-Marie Chagoury School of Medicine, Lebanese American University, Beirut, Lebanon; ORCID: 0000-0002-2980-9933
2. Institut National de Santé Publique d'Épidémiologie Clinique et de Toxicologie-Liban (INSPECT-LB), Beirut, Lebanon
1. Faculty of Nursing and Health Sciences, Notre Dame University-Louaize (NDU), Zouk Mosbeh, Lebanon; ORCID: 0000-0002-6755-4046

***Corresponding Author:**
Samah Tawil
Gilbert and Rose-Marie Chagoury School of Medicine, Lebanese American University, Beirut, Lebanon
Phone: +9619547254, ext. 3466.
Fax: +9611867098
E-mail: samah.tawil@lau.edu.lb

Short Title : Key Trends in AI Applications in Healthcare: A Scoping Review

Investigating the Key Trends in Applying Artificial Intelligence to Health Technologies: A Scoping Review

Abstract

Background: The use of Artificial Intelligence (AI) is exponentially rising in the healthcare sector. This change influences various domains of early identification, diagnosis, and treatment of diseases. **Purpose**: This study presents a scoping review of different studies on the application of AI in the healthcare sector.

Methods: The review included 68 academic studies from different databases. After careful review and data analysis, AI methodologies, benefits and challenges, were summarized.

Results: Our results have shown that AI tools such as deep learning methods and machine learning continue to significantly improve accuracy and timely execution of medical processes. Benefits were discussed from both the organizational and the patient perspective in the categories of diagnosis, treatment, consultation and health monitoring of diseases. However, some challenges may exist, despite these benefits, and are related to data integration and patient safety.

Conclusion: The article examines the present status of AI in medical applications and explores its potential future applications. The findings of this review are useful for healthcare professionals to acquire deeper knowledge on the use of medical AI from design to implementation stage. However, a thorough assessment is essential to gather more insights into whether AI benefits outweigh its risks. Additionally, ethical and privacy issues need careful consideration.

Keywords: artificial intelligence, healthcare, benefits, methodologies, challenges

1. Introduction

Artificial Intelligence (AI) englobes computational technologies that replicate processes associated with human intelligence, including thought, deep learning, adaptation, engagement, and sensory understanding [1,2]. Certain devices, utilizing an interdisciplinary approach, are employed across various fields, particularly in medicine and healthcare [3]. These devices are capable of performing tasks that traditionally require human interpretation and decision-making [4,5]. The integration of AI in medicine dates back to the 1950s, with early attempts by physicians to enhance diagnoses through computer-aided programs [6]. Recent years have witnessed a surge in interest and advancements in medical AI applications, driven by the significantly enhanced computing power of modern computers and the abundance of digital data [7,8]. AI is rapidly transforming the landscape of medicine and healthcare, offering innovative solutions to various challenges [9,10]. It is progressively reshaping medical practices, offering diverse applications in clinical, diagnostic, rehabilitative, surgical, and predictive realms worldwide [11]. AI has become an indispensable tool in various medical applications, revolutionizing traditional practices. Several studies exemplify the integration of artificial intelligence into medicine. Basu et al. (2020) highlights the extensive use of artificially intelligent computer systems inpatient diagnosis and drug development [12]. Another significant study by Buch et al. (2018) explores the current trends and future possibilities of AI in medicine, emphasizing the potential for personalized healthcare through intelligent summarization of vast medical information [13]. Furthermore, AI technology has been employed by researchers across various medical conditions, including the identification of diabetic retinopathy [14], analysis of heart abnormalities [15], and the prediction of risk factors associated with cardiovascular diseases [16]. Additionally, deep learning algorithms

play a role in the detection of pneumonia from chest radiography, demonstrating a sensitivity and a specificity of 96% and 64%, respectively, in contrast to radiologists with rates of 50% and 73% [17]. These studies collectively demonstrate the diverse applications and transformative impact of AI in advancing medical research and patient care. With ongoing developments and increasing adoption, universities in the Middle East and North Africa (MENA) region acknowledged the vital importance of adapting to new technologies and their key role in transforming the region and advancing to the forefront of the digital economy and healthcare. Similarly, Lebanon is actively embracing AI tools marking significant strides in its technological landscape [18-20]. Recently, Lebanon announced the adoption of its first-ever AI Policy, underscoring a commitment to harnessing AI's potential for societal benefit [21-23]. Moreover, there is a growing interest in AI education, with a guide for becoming AI certified in Lebanon, providing insights for enthusiasts and beginners [24,25]. In fact, many Lebanese universities have introduced digital management systems and created new instructional models to enable major educational improvements and enhance learner and instructor experiences. Although the Lebanese advancement in AI technology was slowed by many factors including the COVID-19 pandemic and economic crisis [26-28], Lebanon is still offering a potential avenue for AI integration into different domains with the prospect of transforming healthcare services. It is crucial therefore to systematically document and share information on AI's role in clinical practice, enabling healthcare providers to acquire the knowledge and tools essential for its effective implementation in patient care. This review article delves into the prevalent trends shaping the integration of AI into various medical applications, taking as example the publications of different Lebanese universities between 2020 and 2024 to examine its potential uses, benefits and challenges, while also offering insights into its future development.

Thus, this study seeks to answer the following research questions:

1. What advances has AI brought to the healthcare sector in Lebanon?
2. What are the characteristics of the most recent Lebanese healthcare publications applying AI?
3. What are AI methodologies have been applied for healthcare system in Lebanon?
4. What are the challenges faced by AI applications in health sciences field in Lebanon?

2. Materials and Methods

2.1. Selection Criteria

This study focuses on publications in the field of health and medical sciences extracted by searching Scopus, Web of Science (WoS) and Pubmed, limiting the search to articles and reviews as document types and only considering publications from the time period between 2020 and April 2024. To do this, the following keywords were used in the search strategy: “artificial intelligence [Title/Abstract]) OR (artificial intelligence [MeSH terms]) OR (machine learning [Title/Abstract]) OR (machine learning [MeSH terms]) OR (deep learning [Title/Abstract]) OR (deep learning [MeSH terms]) OR (robot) [Title/Abstract]) OR (robot [MeSH terms]) AND (health) OR (medic) in either the title or abstract as indicated as a Medical Subject Heading (MeSH) term AND “Lebanon” in the affiliations.

All reviewed studies were published in English-language journals or conference proceedings. Studies that were not written in English were excluded. In addition, notes, abstracts, short communications, and letters to the editors were also excluded.

Because numerous observational studies were included, it was challenging to conduct a formal systematic review or meta-analysis. As a result, the choice was made to pursue a scoping review

methodology, with a primary emphasis on observational and qualitative research. Additionally, we offered a brief overview of the main themes found in different types of publications

2.2. Data screening and assessment

A data extraction template was developed to extract all retrieved data. We identified the authors' affiliations from the fields of "affiliations". International collaboration was deemed to exist in an article if any author's affiliation was located outside Lebanon. Citations of the articles in the WOS, SCOPUS and PUBMED databases were extracted in January 2024. To ensure eligibility, extracted results were reviewed by individual reviewers to ensure that the title/abstract includes any aspect of artificial intelligence. The review was divided into a set of phases or steps. The phases followed were: (1) review of previous publications, (2) definition of the inclusion and exclusion criteria, (3) definition of the search strategy, (4) definition of the quality criteria, (5) data extraction, (6) results, and (7) data analysis and report writing.

Extracted data was created in Microsoft excel for article screening, removing duplicate entries, and making appropriate corrections. In a two-phase procedure, two researchers conducted an independent reading of the titles and abstracts of the available articles, and subsequently examined full-text manuscripts to determine eligibility. To ensure methodological rigidity, a third auditor was consulted in case of any discrepancies, and a consensus on article eligibility was reached through rechecking the information. Additionally, a data extraction template for each article that underwent screening for inclusion was filled out. This template included fields for: (1) article title, (2) author names, (3) author count, (4) citations count, (5) journal name, (6) study design, (v7) field of study, (8) authors characteristics such as gender, work status, tenure-track and faculty affiliation, (9) type of the collaboration whether institutional, national (co-authors don't belong to

the same institution but reside in the same country) or international, and (10) Scimago Journal Rank (SJR) and quartile (Q) of the journal where Q1 comprises the quarter of the journals with the highest values, Q2 the second highest values, Q3 the third highest values and Q4 the lowest values. Finally, the authors evaluated the methodological quality of the selected articles and classified them based on the type of AI used, and the purpose for which it was implemented. The composition of the review adhered to the PRISMA guidelines designed for scoping reviews [29]

2.3. Quality assessment

Quality assessment of the included studies was completed according to the Grading of Recommendations, Assessment, Development and Evaluation (GRADE) tool [30]. Every potential source of bias was assessed and categorized as either very low, low, intermediate, high, or unclear based on the specific study design: randomized trials, downgraded or upgraded observational studies, and case series/reports (considered very low).

2.4. Data Abstraction and synthesis

Main findings from the studies were reviewed using descriptive and analytical methods based on different variables outcomes. Statistical analysis was performed using SPSS version 29 (IBM SPSS Software, Chicago, IL, USA). Continuous measures were summarized using either means and standard deviations or medians and interquartile ranges, depending on appropriateness. Categorical measures were summarized using frequencies and percentages. To compare the number of citations among different medical fields, ANOVA test was used. A *p-value* ≤ 0.05 was considered to be statistically significant.

2.5. Patient and Public involvement

Since this review did not involve human participants, there was no requirement to seek informed consent

3. Results

3.1 Study selection

A total of 2056 publications were identified through initial searches out of which 1340 studies focused on medical or health sciences. A first selection process involved the removal of studies not related to AI. After conducting an initial screening based on title and abstract, we included 270 contributions for a full-text review. Another selection process was performed to remove duplicates or letter to editors, or erratum or corrections to previous publications. After reading the full articles, another 192 articles were considered insignificant and were removed, which resulted in 78 remaining articles related to the research question. The most common reasons for excluding some journal articles after full-text review were that some of them were not pertinent or relevant to the research question and others didn't include AI as a primary objective. A PRISMA flow-diagram is presented in Fig. 1 to illustrate the study selection process.

Fig 1. PRISMA flow diagram of studies selection process

(Caption: Process of inclusion and exclusion of studies)

3.2 Publications' characteristics and quality assessment

A total of 68 publications related to the application of AI in the medical or healthcare field indexed in Scopus, WoS, and PubMed databases between 2020 and 2023 were included in our review. Fig. 2 displays the percentage of publications per year. The number of publications showed a steady rise from 2020 to 2023, peaking at its highest point (44.1%) in 2023 and an exponential increase in number is expected in 2024. Most of the publications were the results of a collaborative work with international universities (92.1%). Most of the authors were affiliated to the Lebanese American University (52.9%), followed by the American University of Beirut (25.0%).

Fig 2. Percentage of AI in health studies across the years

(Caption: Percentages of health studies that apply AI across the years)

The vast majority of all publications were modeling studies (41.2%) or descriptive/narrative reviews (29.4%). Among all publications, the most abundant health-related subject of interest was related to cancer diagnosis and management (25%) followed by cardiology (20.6%), then mental health diagnosis (10.3%) (Fig.3).

Fig 3. Main areas of AI application

(Caption: percentage of health-related subject that apply AI)

All the studies were characterized by multiple authorships. The average number of authors per study was 6.8±2.9. More details about the characteristics of publications are summarized in Table 1. The GRADE evaluation form was employed to assess the primary outcomes of the included studies. The quality of evidence for interventional studies was categorized as high, while observational studies were rated as medium to low in terms of quality. Literature reviews were regarded as having a very low level of evidence quality

**Table 1. Characteristics of the publications**		
	**Number (N)**	**Percentage (%)**
**Authors' count**		
< 2	4	5.9
≥ 2	64	94.1
**Work Collaboration**		
Local	3	4.4
National	3	4.4
International	62	91.2
**Study Design**		
Invitro/in-vivo/in-silico	7	10.3
Cohort	3	4.4
Cross-sectional	5	7.4
Modeling	28	41.2
Review (general)	20	29.4
Systematic review and Meta-analysis	5	7.4
**Academic Institution**		
American University of Beirut	17	25
Lebanese American University	36	52.9
Lebanese University	5	7.4
Beirut Arab University	3	4.4
Saint Joseph University	7	10.3
Others	3	4.4

3.3. Characteristics of the journals and citation numbers

As detailed in Table 2, all of the publications authored by all scholars appeared in peer-reviewed journals. The majority of the publications were published in SJR Q1 journals (66.7%)

while 19 papers (28.8%) belonged to Q2 journals. Only three publications were submitted to journals of lower SJR rank. The most repetitive journal is Scientific Reports followed by Diagnostics.

**Table 2. Characteristics of the journals**		
	**Number (N)**	**Percentage (%)**
**Journals per quartile**		
Q1	45	66.2
Q2	20	29.2
Q3	3	4.4
**Citations**		
0	16	26.7
1	8	13.3
2-10	24	40.0
>10	12	20.0
**Most repetitive journals**		
Scientific Reports	9	13.2
Diagnostics	4	5.9
Sensors	2	2.9
Journal of Vascular Surgery	2	2.9
Studies in Health Technology and Informatics	2	2.9

*Q: Journal Quartile

The majority of the studies were cited at least once (73.3%) while only 26.7% received no citations. All publications were cited a total of 454 times, the mean citation number was 7.57±12.31, and 40% of the publications were cited 2 to 10 times. As shown in Fig. 4, immunology field received the highest number of citations (mean±SD: 11.17 ±11.8) followed by cardiology (mean±SD: 8.75±16.36). However, this difference in the number of citations didn't reach statistical significance (p = 0.092).

Fig 4. Differences in the average number of citations among different medical fields

(Caption: Mean citation numbers across different fields of study)

3.4. Framework of Research Classification

All included studies were organized into three dimensions: AI methodologies, benefits, and challenges. All articles were attributed an AI methodology and were aggregated under the benefits dimension while only 7 studies were aggregated under the challenges dimension.

Table 3 shows the classification framework related to AI areas/methodologies that were discussed in the selected studies and contains specific categories for this dimension, such as data or multimedia. In addition, in each category, relevant factors were identified that defined the category. Table 4 illustrates the classification framework related to both AI enabled healthcare benefits and challenges associated with the use of AI in different healthcare fields and involves specific categories for this dimension, such as challenges related to data integration and patient safety.

3.4.1. AI Methodologies

In the context of applying AI in healthcare, methodology refers to the procedures or areas healthcare institutions implement to utilize AI. The two main AI areas are related to either data processing or multimedia processing. The data processing includes both Deep Learning Methods (DLM) or Machine Learning (ML); and the multimedia processing can be divided into AI devices/imaging or video processing or virtual reality [31].

A. Data processing

Of the selected studies, 75% (n=51) applied AI technology based on data processing models, of which 66% (n=34) applied the DLM and the other 34% were based on ML. The main therapeutic field that used DLM as the main AI tool is the oncology field (n=11). Among these studies, four were related to brain tumors [32-35], and the others discussed its use in the context of different cancers such as prostate [36], skin [37], breast [38], bone [39], pancreatic [40], and colon [41] or in the general context of prediction of cancer metastasis [42]. Also, six studies from the cardiology studies discussed the use of these DLM as an AI tool for diagnosis and detection of different cardiovascular diseases [43-48] followed by five studies from the infectiology domain [49-53], mental health [54,55], gastroenterology [56], neurology [57,58] and other various sectors [59-65]. As for AI-based ML, it was most commonly mentioned in studies from the cardiology unit (n=6) [66-71] followed by mental studies [72-74] then cancer studies [75,76] in addition to diverse other domains [77-82]. More details on AI data processing tools can be found in Table 3.

Table 3. AI methodologies applied in the healthcare setting

Study		Date	Medical area	AI Category	AI Area	Summarized content
Gumaei et al.	36	2021	Oncology	Data processing	Deep learning methods /modeling	Use of microarray gene expression for prostate cancer diagnosis
Zafar et al.	37	2023				Use of deep learning techniques for skin lesion analysis and cancer detection
Kumar et al.	32	2023				Use of deep neural network and transfer learning for brain tumor classification
Ghanem et al.	33	2023				Use of deep learning approaches for glioblastoma prognosis
Ullah et al.	35	2024				Use of novel optimal framework of residual blocks and stacked autoencoders for multimodal brain tumor classification
Zaylaa et al.	38	2024				Use of breast mass images, machine learning, and regression models for breast cancer diagnosis
Dhasmana et al.	40	2023				Use of integrative big transcriptomics data analysis in the detection of MUC13 in pancreatic cancer
Yagin et al.	42	2023				Use of explainable artificial intelligence in cancer metastasis prediction
Halabi et al.	34	2023				Use of AI in unveiling RNA biomarkers for the diagnosis and prognosis of meningioma
Rajinikanth et al.	41	2023				Use of deep-learning framework for colon histology classification
Magdy et al.	39	2023				Bone metastasis detection using ai optimization algorithm
Rammal et al.	75	2022			Machine Learning	Use of machine learning techniques for prostate cancer diagnosis
Ghaith et al.	76	2023				Use of machine learning to predict prognosis associated with glioma
Lucchetti et al.	84	2023		Multimedia processing	AI devices/Imaging processing	Application of smart nano-sized extracellular vesicles for gastrointestinal cancer therapy
Hage Chehade et al.	85	2022				Use of ai medical imaging for lung and colon cancer classification
Felefly et al.	83	2023				MRI-radiomic quantum neural network to differentiate between large brain metastases and high-grade glioma
Atat et al.	97	2022			Virtual reality	Use of 3d modeling in cancer studies
Walsh et al.	43	2020	Cardiology	Data processing	Deep learning methods /modeling	Description of a speckle-tracking strain-based artificial neural network model to differentiate cardiomyopathy type
Ahmad et al.	44	2020				Comparison of ai-based algorithms for the identification of depressed right ventricular function with 2-d echocardiography

Helwan et al.	45	2024				Description of conventional and deep learning methods in heart rate estimation from RGB face videos
Guldogan et al.	46	2023				Description of AI approach for the prediction of angina pectoris
Mitu et al.	47	2024				Use of AI explainable ensemble learning to predict a stroke
Li et al.	48	2024				Development and evaluation of a prediction model for peripheral artery disease
Barakett-Hamade et al.	66	2021				Comparison of machine learning-derived low-density lipoprotein cholesterol to standard closed form equations
Li et al.	69	2024				Use of machine learning to predict outcomes of endovascular abdominal aortic aneurysm repair
Li et al.	67	2023			**Machine Learning**	Use of machine learning to predict outcomes of open revascularization for aortoiliac occlusive disease
Li et al.	68	2023				Use of machine learning to predict major adverse cardiovascular events following carotid endarterectomy
Hammoud et al.	71	2023				Use of machine learning to predict incomplete occlusion of intracranial aneurysms treated with flow diverters
Li et al.	70	2024				Use of machine learning to predict outcomes following lower extremity open revascularization
Serhal et al.	90	2022		**Multimedia processing**	**AI devices/Imaging processing**	Overview on prediction, detection, and classification of atrial fibrillation using wavelets and AI on electrocardiogram
Moshawrab et al.	91	2023			**AI devices/video processing**	Use of smart wearables for the detection of cardiovascular diseases
Ghazi et al.	77	2024	**Nephrology**	**Data processing**	**Machine Learning**	Comparison of biomarkers to machines in predicting acute kidney injury
Alnazer et al.	89	2021		**Multimedia processing**	**AI devices/Imaging processing**	Medical image processing for the evaluation of chronic kidney disease
Helwan et al	49	2021				Comparison of radiology to deep convolutional neural network in diagnosing COVID-19
Rashid et al.	50	2023				Use of using deep learning method for white blood cell image analysis and infection detection
Tarek et al.	51	2023	**Infection**	**Data processing**	**Deep learning methods /modeling**	Use of using deep learning method for covid-19 death prediction
Amin et al.	52	2024				Use of generalized normal distribution optimization for malaria classification
Ngugi et al.	53	2024				Use of computational deep learning for crop disease detection
Saleh et al.	98	2020			**Virtual reality**	Description of 3D model to study respiratory syncytial virus infections

Acharya et al.	88	2022		**Multimedia processing**	**AI devices/Imaging processing**	Use of a deep learning normalization-free network model for tuberculosis detection and classification
Javed et al.	54	2023	**Mental health**	**Data processing**	**Deep learning methods / modeling**	Description of AI-empowered cognitive health assessment
Jaber et al.	55	2022				Use of AI model based on physiological measurements to predict stress occurrence
Qasrawi et al.	72	2022			**Machine Learning**	Use of machine learning techniques for the prediction of depression and anxiety in pregnant and postpartum women during the COVID-19
Mahalingam et al.	73	2023				Use of machine learning to predict anxiety
El Morr et al.	74	2024				Use of machine learning models to assess Lebanese university students' depression, anxiety, and stress during covid-19
Boulos et al.	92	2021		**Multimedia processing**	**Virtual reality**	Description of digital mental health
Kabbara et al.	93	2022				Description of electroencephalogram predictive model for detecting the severity of major depressive disorder
Ghanem et al.	78	2023	**Orthopedics**	**Data processing**	**Machine Learning**	Description of the limitations of machine learning models used in spine surgery
Yammine et al.	96	2022		**Multimedia processing**	**Virtual reality**	Description of the outcomes of the use 3d printing models in fracture management
Ramzan et al.	56	2023	**Gastroenterology**	**Data processing**	**Deep learning methods/modeling**	Use of deep learning models for the classification of gastrointestinal tract disorders
Dangi et al.	86	2023		**Multimedia processing**	**AI devices/Imaging processing**	Use of nanotechnology impacting probiotics and prebiotics in the composition of nutraceuticals
Hammoud et al.	79	2022	**Gynecology**	**Data processing**	**Machine Learning**	Survey of the use of machine learning models to predict maternal and newborn healthcare providers' perception of safety during the covid-19 pandemic
Jerbaka et al.	95	2022		**Multimedia processing**	**Virtual reality**	Description of the outcomes of robotic and laparoscopic surgery for benign gynecological disease
Hallal et al.	59	2021	**Genetics**	**Data processing**	**Deep learning methods/modeling**	Use of a deep learning framework to predict histone marks in open chromatin regions at time-points with missing chip-seq datasets
de Brevern et al.	60	2022				Description of the current status of post-translational modifications (PTMs) structural databases
Ali et al.	57	2024	**Neurology**	**Data processing**	**Deep learning methods/modeling**	Use of deep neural network for detection of Parkinson's disease
Voigtlaender et al.	58	2024				General description of AI implications in neurology
Chedid et al.	80	2022			**Machine Learning**	Description of the development of an automated machine learning pipeline for the detection of Alzheimer's disease
Hussain et al.	61	2023	**Dermatology**	**Data processing**	**Deep learning methods/modeling**	Description of the use of deep neural networks and algorithms for multiclass skin lesion localization and classification

Bibi et al.	62	2023				Description of the use of fine-tuned deep models' information fusion and best feature selection for multiclass skin lesion
Al-Sheikh et al.	63	2023	**Pneumology**	**Data processing**	**Deep learning methods/modeling**	Use of deep learning architecture for classifying lung diseases
Dasegowda et al.	87	2023		**Multimedia processing**	**AI devices/Imaging processing**	Use of AI in the advancement of suboptimal chest radiography
Askin et al.	64	2023	**General**	**Data processing**	**Deep learning methods/modeling**	Artificial intelligence applied to clinical trials: opportunities and challenges
Saab et al.	81	2022			**Machine Learning**	Development and validation of a biomarker-based machine learning model to predict all-cause clinical deterioration in general wards
Saab et al.	82	2020				Use of different machine learning algorithms for classifying adverse-event related 30-day hospital readmissions and patient safety
Malik et al.	99	2023		**Multimedia processing**	**Virtual reality**	Description of emerging applications of nanotechnology in healthcare and medicine
Malik et al.	94	2023	**Dentistry**	**Multimedia processing**	**Virtual reality**	Emerging applications of nanotechnology in dentistry
Venkatapathappa et al.	65	2024	**Opthtalmology**	**Data processing**	**Deep learning methods/artificial intelligence**	Description and use of transformative AI in ocular genetics

B. *Multimedia processing*

As shown in Table 3, 25% of the included studies applied AI technology based on multimedia processing models, of which 53% (n=9) discussed the use of AI devices, imaging or video processing, and 47% applied virtual reality to improve diagnosis or detection of certain diseases. Studies from the oncology field (n=3) predominantly used AI devices to detect and diagnose some types of cancers such as brain [83], gastric [84] or colon [85]. Other fields that employed the same AI tool belonged to gastroenterology to evaluate the use of nanotechnology in the composition of nutraceuticals [86], pulmonary chest radiography [87], detection and classification of infectious diseases [88], kidney diseases [89] as well as prediction, detection, and classification of cardiovascular diseases like atrial fibrillation [90,91]. Virtual reality played an important role in mental health studies particularly in describing digital mental health procedures and models for detection of depression [92,93]. Moreover, it was also used as an emerging application in dentistry [94], gynecological [95], orthopedic procedures [96] and cancer detection [97] in addition to infectious disease [98] as well as general medical research [99].

3.4.2. Benefits of AI

This aspect refers to the achievable benefits gained through AI utilization. These include benefits to individuals, such as automated decision-making, patient monitoring and prognosis, early diagnosis, and process simplification and therapeutic management. Additionally, they include benefits to organizations like optimizing workflow management, improving performance, and ensuring data availability.

A. Benefits to individuals

The exploration of the included studies showed that there is a growing interest in exploring the potential of AI-based support systems in improving early disease diagnosis. This is highlighted by the fact that many examined studies concentrated on this aspect (n=14). Of these, the most commonly repetitive therapeutic field was related to cancer [36,39,40,75] followed by mental health (depression and anxiety) [72-74] and other infectious diseases such as tuberculosis [88], crop disease [53] and coronavirus detection [45]. Moreover, a very recent study conducted by Ghazi et al. investigated the use of AI machines to predict acute kidney injury [77].

Furthermore, the use of AI-powered simulations to enhance healthcare decision-making abilities were discussed in different studies across diverse therapeutic domains including gynecology [79], cardiology [67] and cognitive health [54]. Similarly, other studies concluded that AI-based resources have the potential to improve patient outcomes and prognosis in various oncological, cardiovascular and immunological studies [33,42,51,67,69,76]. Likewise, another AI benefit - process simplification - has been proven in 14.7% (n=10) of the studies. This implication was seen in different medical domains including mainly cardiology imaging [43,68,70,71,91] and physiological measurements of mental health diseases [55,93]. In the same manner, AI-assisted tools, such as 3D printing models and theragnostic applications, have shown to offer benefits in therapeutic management and clinical outcomes, as evidenced by studies conducted by Yammin et al. (2022) [84] and Lucchetti et al (2024) [96].

Table 4. AI enabled benefits and challenges in the healthcare setting

Dimension	Category	Type	Studies
**Benefits**	**Individual**	**Disease diagnosis**	Rammal et al., 2022 [75] ; Dhasmana et al., 2023 [40] ; Ahmad et al., 2020 [44] ; Acharya et al., 2022 [88] ; Qasrawi et al., 2022 [72] ; Mahalingam et al., 2023 [73] ; El Morr et al., 2024 [74] ; Chedid et al., 2022 [80] ; Mitu et al., 2024 [47] ; Ghazi et al., 2024 [77] ; Helwan et al., 2021 [49] ; Ngugi et al., 2024 [53] ; Gumaei et al., 2021 [36] ; Magdy et al., 2023 [39]
		**Patient monitoring/prognosis**	Ghanem et al., 2023 [33] ; Ghaith et al., 2023 [76] ; Tarek et al., 2023 [51] ; Yagin et al., 2024 [42] ; Li et al., 2024 [67] ; Li et al., 2023 [69]
		**Decision-making**	Javed et al., 2023 [54] ; Yammine et al., 2022 [96] ; Li et al., 2024 [48] ; Hammoud et al., 2022 [79]
		**Process simplification**	Walsh et al., 2020 [43] ; Moshawrab et al., 2023 [91] ; Li et al., 2023 [70] ; Hammoud et al., 2023 [71] ; Li et al., 2024 [68] ; Jerbaka et al., 2022 [95] ; Amin et al., 2024 [52] ; Jaber et al., 2022 [55] ; Kabbara et al., 2022 [93] ; Felefly et al., 2023 [83]
		**Therapeutic management**	Lucchetti et al., 2023 [86]
	**Organization**	**Performance improvement**	AlNazer et al., 2021 [89] ; Hussain et al., 2023 [61] ; Bibi et al., 2023 [62] ; Ramzan et al., 2023 [56] ; Hallal et al., 2021 [59] ; Saleh et al., 2020 [98] ; Rashid et al., 2023 [50] ; Ali et al., 2024 [57] ; Saab et al., 2020 [82] ; Al-Sheikh et al., 2023 [63] ; Saab et al., 2022 [81] ; Hage Chehade et al., 2022 [85] ; Zafar et al., 2023 [37] ; Kumar et al., 2023 [32] ; Halabi et al., 2023 [34] ; Rajinikanth et al., 2023 [41] ; Ullah et al., 2024 [35] ; Zaylaa et al., 2024 [38] ; Barakett-Hamade et al., 2021 [68] ; Helwan et al., 2024 [45] ; Guldogan et al., 2023 [46]
		**Data availability**	Askin et al., 2023 [64] ; Voigtlaender et al., 2024 [58] ; Venkatapathappa et al., 2024 [65] ; Dasegowda et al., 2023 [87] ; Ghanem et al., 2023 [78] ; Malik et al., 2023 [99] ; Malik et al., 2023 [94] ; Atat et al., 2022 [97] ; Serhal et al., 2022 [90] ; de Brevern et al., 2022 [60]
		**Workflow management**	Dangi et al., 2023 [86] ; Boulos et al., 2021 [92]
**Challenges**	**Data Integration**	**Data availability**	Mitu et al, 2024 [47] ; Ngugi et al., 2024 [53]
		**Digitalization**	Askin et al., 2023 [64] ; deBrevern et al., 2022 [60]
	**Patient Safety**	**Data errors**	Voigtlaender et al., 2024 [58] ; Ghanme et al., 2023 [78]
		**Decision errors**	Malik et al., 2023 [99]

B. Benefits to organizations

Organizations use AI applications and tools to provide a better workflow management [86,92], overcome data availability and integration [58,60,64,65,78,87,90,94,97,99] and improve organizational improvement of different medical departments such as cancer imaging and

diagnosis [32,34,35,37,38,41,85], cardiovascular disease prediction and management [45,46,66], dermatological lesions recognition [61,62], or infection detection [50,98]. Moreover, in his prior studies, Saab et al. described the use of ML for general therapeutic purposes such as the early prediction of all-cause clinical deterioration [81] and the classification of adverse-event related 30-day hospital readmissions [82]. More details on the benefits of AI applications are summarized in Table 4.

3.4.3. Challenges of AI

As detailed in Table 4, a number of challenges may deter organizations from using AI. Nevertheless, the number of studies focusing on AI challenges has been the lowest in the past ten years. The most occurring challenges in this review were related to data integration and/or patient safety.

Challenges related to data integration consist of data availability in cardiology and immunology units [47,53] as well as data digitization and consolidation in genetics and medical research field [60,64]. AI Challenges, from the patient's perspective, are related to decision errors [99] and data errors in both neurologic and orthopedic surgeries [58,78].

4. Discussion

4.1. Principal findings

AI is gaining significant attention across various fields, including medicine. The purpose of this review was to gather and summarize the existing information regarding the use of artificial intelligence in the healthcare setting in Lebanon. The AI models are mainly theoretical, utilizing

automation or optimization technologies. They are mostly designed for clinical care and diagnostic purposes. Most of them are intended to assist human decision-making and are conceptual lacking empirical evidence [100].

Most of the studies have been recently published (2020-2024) and are characterized by their prediction/modeling design which is expected given the timeline and the future perspective of AI in healthcare research. To add, many papers have multiple authors, with international collaboration being the most prevalent form of authorship. This is essential due to the topic's significance, requiring expertise from various domains given its novelty. This is also consistent with other prior publications, confirming that research collaboration adds benefits for both the researchers and the organizations and enhances the quality of research resulting in higher numbers of scholarly output [101].

In terms of data analysis, most of the included journals are published in Q1 reputable journals which are optimal locations for the publication of scientific articles focused on a specialized topics like AI. These journals deal mainly with healthcare, medical information systems, and applications such as informatics, and machine learning. Additionally, in terms of citation numbers, it was shown that most of these studies possess multiple citations, and the mean number of citations was not significant across fields - which emphasizes the impact of this productive research on other research works regardless of the therapeutic domain.

Upon reviewing the pertinent studies, our results summarize the main methodologies of the AI areas implicated in the healthcare setting as well as the benefits and challenges associated with the use of AI in enhancing healthcare. The effectiveness of AI, in medical care, is influenced by the type of intelligence utilized and its applications [102,103]. Accordingly, this review, similar to other previous studies, found that ML models are commonly used in diagnostic support systems

[104, 105]. The popularity of this AI type is mainly due to its efficiency and cost-effectiveness in performing human tasks [106]. Similarly, our results have shown that the advancement of AI in healthcare is mainly focused on diagnosing and predicting diseases such as cancer, cardiovascular, infectious diseases, and various chronic neurological disorders and skin conditions. This focus helps healthcare professionals spend more time with patients, use a holistic care approach, and improve patient satisfaction [107]. Moreover, the deep learning sequence is also considered as an essential improvement of different strategies utilized to upgrade healthcare practice [108]. With the use of modern computational methods and computer learning, more data would become available, which could give insights into many different medical and healthcare practices [109]. Our study has shown that AI makes it easier to turn data into concrete and predictable observations to improve disease diagnosis, patient monitoring, decision-making, and deliver high-quality therapeutic management in many different fields such as oncology, cardiology, and even mental or neurologic diseases. It has also shown that, in a similar perspective to previous research using these computational tools, healthcare professionals may use data not only to describe actual events but also to predict future outcomes and foster future possibilities to enhance organizational performance and enhance workflow management [110, 111]. On the other hand, other AI technologies, including robotic process automation and physical robots, have demonstrated their effectiveness in some specific studies [112]. These AI solutions enhance disease identification and management, as well as adherence to treatment protocols. However, based on the results of our review, it can be inferred that these AI types are less familiar in the Middle Eastern region compared to more prevalent technologies like DLP and ML.

In addition to the benefits on the organizational level, one of the notable benefits of AI techniques is the potential support for comprehensive health services management on the individual level. For

instance, and as mentioned before, an AI system can offer health professionals continuous updates on medical issues to improve patient safety, and promote treatment efficacy [113,114]. Other AI tools permit the integration of patient information tools and the generation of outcome predictions [115, 116]. AI is effective in analyzing large datasets and generating innovative, relevant solutions for health practitioners, enhancing patient care, diagnosis, and treatment options which usually require significant time and effort. By making automation easier and more available with minimal human intervention, AI can even surpass human performance in certain medical scenarios, such as radiology, cardiology, and tumor detection [117].

On the contrary of the previous studies which primarily focused on the positive aspects of AI like prediction and decision-making, our analysis explores the potential challenges faced by AI application in the healthcare setting in addition to the future uses and benefits of technology in health service management. For example, Mitu et al. reported the challenges regarding data integration and availability [47] and Ghanem (b) et al. reported AI decision errors faced in orthopedic surgery [78]. Likewise, some authors highlighted these difficulties which hinder achieving clinically relevant results [118]. Sometimes the applied algorithm may be inappropriate for the data, or the data may still lack enough reliability for use in classification algorithms like neural networks and decision trees. Several previous studies have also demonstrated these AI - related challenges and possible decision-making problems in the health domain and discussed their solutions [119, 120, 121] which explains why major AI companies are actively identifying priority areas, opportunities, and recommendations to address these concerns in healthcare practice [122, 123].

4.2. Limitations

Despite the results and findings obtained, the presented article has several limitations. Although positive results related to the use of AI were shown for most of the analyzed types, more studies are necessary to consider the current limitations of these systems and to meet the needs of professionals in the development of AI-based systems.

Moreover, the application of AI in healthcare, particularly in low socio-economic countries like Lebanon, is still developing and lacks substantial evidence. This might be due to the predominance of observational and descriptive studies, which exhibit a great heterogeneity in AI types and settings. Therefore, generalizations of the proposed results should be done cautiously. Furthermore, carrying out a meta-analysis or systematic review was not considered in this review due to the heterogeneity in the study variables and designs. To add, the concept of AI remains vague. While we tried to mitigate this limitation by using various MESH terms in our search strategy, the resulting findings may still be general and could have excluded some relevant studies.

4.3. Future implications and recommendations

By using AI, healthcare professionals can benefit from a better understanding of new medical devices and software that ensure faster medical care, reduced workloads, and more accurate treatments which improve patients' overall quality of life. The advantages of AI in healthcare extend beyond medical uses by reducing physical, emotional, and workload stress, especially for exhaustive or repetitive tasks. Based on our results, it is suggested that an analysis would be useful in assessing the total cost of these AI technologies taking into account their importance in ensuring the modernization of healthcare organizations. This suggestion is specifically important in countries with low socio-economic profile such as Lebanon, where

infrastructure of AI services is still lacking [124]. On the other hand, it would be interesting to assess additional benefits and drawbacks associated with the use of AI technologies in healthcare. For this reason, it would be helpful to carry out a comparative quantitative analysis between countries engaged in this type of versus those that are not, in order to enhance our knowledge and expand the access of healthcare organizations to AI-based technologies. The improvement of the ethical and legal standards for the use of AI will facilitate its adoption in the society while acknowledging potential risks. The findings will be useful for healthcare professionals as well AI engineers, developers, and researchers to improve the circulation and utilization of medical AI. Therefore, governments can significantly play an important role in supporting empirical research and practical applications through the development and dissemination of an AI-specific implementation framework to improve and adapt policies to secure patient data to promote confidentiality. This framework would address key aspects such as building trust, creating explainable solutions, and tackling ethical concerns regarding issues regarding privacy and data protection associated with AI.

5. Conclusion

Our scoping review compiles the available evidence on various AI-based support systems that can be integrated into healthcare practice. It gives an overview of the most used methodologies in the AI in healthcare setting as well as their benefits and challenges in a Middle eastern country. Further, despite the few challenges that exist with this type of technology, the results of the different types of AI are promising in the healthcare setting. However, it is still essential to discover whether these benefits outweigh the risks/challenges related to its use. It remains also essential to

consider ethical, legal and privacy concerns as well as ensure that AI is used to enhance the role of healthcare professionals rather than replace them.

Statement and Declarations

- **Ethics approval and consent to participate:** Not applicable
- **Consent for publication:** Not applicable
- **Availability of data and materials:** The datasets used and/or analyzed during the current study are available from the corresponding author on reasonable request.
- **Competing interests:** The authors declare that they have no competing interests
- **Funding:** Not applicable
- **Authors' contribution:** ST: conceptualization, methodology, analysis, writing- Original draft preparation. SM: Validation, Writing- Reviewing and Editing
- **Acknowledgements**: Not applicable

References

1. Tagliaferri, S.D., Angelova, M., Zhao, X., Owen, P.J., Miller, C.T., Wilkin, T., et al. Artificial intelligence to improve back pain outcomes and lessons learnt from clinical classification approaches: three systematic reviews. NPJ Digit Med.3:93 (2020). doi:10.1038/s41746-020-0303-x

2. Khan, A., Asif, A., Laghari, A., Rashid, M., Li, H., Javed, A., et al. Artificial intelligence and blockchain technology for secure smart grid and power distribution automation: a state-of-the-art review. Sust Ener Tech Ass.57:103282. (2023). doi:10.1016/j.seta.2023.103282.

3. Mathkor, D.M/, Mathkor, N., Bassfar, Z., Bantun, F., Slama, P., Ahmad, F., Haque, S. Multirole of the internet of medical things (IoMT) in biomedical systems for managing smart healthcare systems: An overview of current and future innovative trends. J Infect Public Health. 4:559-572. (2024). doi: 10.1016/j.jiph.2024.01.013

4. Panch, T., Szolovits, P., Atun, R. Artificial intelligence, machine learning and health systems. J Glob Health. 8(2):020303. (2018)

5. Barnawi, A., Gaba, S., Alphy, A., Jabbari, A., Budhiraja, I., Kumar, V., et al. A systematic analysis of deep learning methods and potential attacks in internet-of-things surfaces. Neur Comput App. 35(25): 18293-18308. (2023) doi:10.1007/s00521-023-08634-6

6. Burton, R.J., Albur, M., Eberl, M., Cuff, S.M. Using artificial intelligence to reduce diagnostic workload without compromising detection of urinary tract infections. BMC Med Inform Decis Mak.19(1):171. (2019).

7. Tout, H., Kara, N., Talhi, C., Mourad A. Proactive machine learning-based solution for advanced manageability of multi-persona mobile computing. Comp Elect Eng.80:10649. (2019). doi:10.1016/j.compeleceng.2019.106497

8. Seifeddine, K., Gandomi, A. ISMSI '23: Proceedings of the 2023 7th International Conference on Intelligent Systems, Metaheuristics & Swarm Intelligence:152–158. (2023). Association for Computing Machinery, New York, NY, USA.. doi.org/10.1145/3596947.3596963

9. El Naamani, K., Musmar, B., Gupta, N., Ikhdour, O., Abdelrazeq, H., Ghanem, M., et al. The Artificial Intelligence Revolution in Stroke Care: A Decade of Scientific Evidence in Review. World Neurosurg.184:15–22. (2024). https://doi.org/10.1016/j.wneu.2024.01.012

10. Saeed, U., Insaf, R.A., Piracha, Z.Z., Tariq, M.N., Sohail, A., Abbasi, U.A., et al. Crisis averted: a world united against the menace of multiple drug-resistant superbugs -pioneering anti-

AMR vaccines, RNA interference, nanomedicine, CRISPR-based antimicrobials, bacteriophage therapies, and clinical artificial intelligence strategies to safeguard global antimicrobial arsenal. Front microb.14:1270018.(2023). https://doi.org/10.3389/fmicb.2023.1270018

11. Altalbe, A., Javed, A.R. Privacy preserved brain disorder diagnosis using federated learning. Comp syst Sci Eng. 47(2):2187- 2200. (2023) . https://doi.org/10.32604/csse.2023.040624

12. Basu, K., Sinha, R., Ong, A., Basu, T. Artificial Intelligence: How is it changing medical sciences and its future? Indian J Dermatol. 65(5):365-370. (2020). doi: 10.4103/ijd.IJD_421_20.

13. Buch, V.H., Ahmed, I., Maruthappu, M. Artificial intelligence in medicine: current trends and future possibilities. Br J Gen Pract. 68(668):143-144. (2018). doi: 10.3399/bjgp18X695213.

14. Li, S., Zhao, R., Zou, H. Artificial intelligence for diabetic retinopathy. Chin Med J (Engl).135(3):253–60. (2021). https://doi.org/10.1097/CM9.0000000000001816.

15. Umer, M., Aljrees, T., Karamti, H., Ishaq, A., Alsubai, S., Omar, M., Bashir, A.K., Ashraf, I. Heart failure patients monitoring using IoT-based remote monitoring system. Sci Rep.13(1):19213. (2023). doi: 10.1038/s41598-023-46322-6

16. Alfaras, M., Soriano, M.C., Ortín, S. A fast machine learning model for ECG-based Heartbeat classification and arrhythmia detection. Front Phys.7. (2019). https://doi.org/10.3389/fphy.2019.00103

17. Becker, J., Decker, J.A., Römmele, C., Kahn, M., Messmann, H., Wehler, M., et al. Artificial intelligence-based detection of pneumonia in chest radiographs. Diagnostics. 12(6):1465. (2022). https://doi.org/10.3390/diagnostics12061465.

18. Abdulkadir, R.A., Hasan, M.K., Islam, S., Gadekallu, T., Pandey, B., Safie, N., et al. Novel residual hybrid machine learning for solar activity prediction in smart cities. Earth Sci Inform.16, 3931–3945. (2023). https://doi.org/10.1007/s12145-023-01130-4

19. Bouraima, M.B., Ayyıldız, E., Badi, I., Özçelik, G., Yeni, F.B., Pamucar, D. An integrated intelligent decision support framework for the development of photovoltaic solar power. Eng App Art Int.127:107253. (2024). doi:10.1016/j.engappai.2023.107253

20. Aqeel, I., Khormi, I.M., Khan, S.B., Shuaib, M., Almusharraf, A., Alam, S., Alkhaldi, N.A. Load Balancing Using Artificial Intelligence for Cloud-Enabled Internet of Everything in Healthcare Domain. Sensors. 23(11):5349. (2023). https://doi.org/10.3390/s23115349

21. Rajhi, W., Ibrahim, A., Mourad, A., Boujelbene, M., Fujii, M., Elsheikh, A. Prediction of milled surface characteristics of carbon fiber-reinforced polyetheretherketone using an optimized

machine learning model by gazelle optimizer. Measurement. 222:113627. (2023). doi:10.1016/j.measurement.2023.113627

22. Shang, Y., Yang, Q., Pu, Y., Taghizadeh-Hesary, F. Employing artificial intelligence and enhancing resource efficiency to achieve carbon neutrality. Res Pol.88:104510. (2024). doi:10.1016/j.resourpol.2023.104510

23. Zaidi, A., Samuel-Soma, M., Ajibade, M., Bekun, F. New insights into the research landscape on the application of artificial intelligence in sustainable smart cities: a bibliometric mapping and network analysis approach. Int J En Econ Policy.13(4):287-299. (2023). https://doi.org/10.32479/ijeep.14683

24 Dakakni, D., Safa, N. Artificial intelligence in the L2 classroom: Implications and challenges on ethics and equity in higher education: A 21st century pandora's box. Comp Educ: Artif Int. 5:100179. (2023). doi:10.1016/j.caeai.2023.100179

25. Yenduri, G., Kaluri, R., Rajput, D.S., Lakshmanna, K., Gadekallu, T.R., Mahmud, M., et al. From assistive technologies to metaverse—technologies in inclusive higher education for students with specific learning difficulties: A review. Institute of Electrical and Electronics Engineers (IEEE). (2023). doi:10.1109/access.2023.3289496

26. Koweyes, J., Salloum, T., Haidar, S., Merhi, G., Tokajian, S. COVID-19 pandemic in Lebanon: one year later, what have we learnt? mSystems.6(2):e00351-21. (2021). doi: 10.1128/mSystems.00351-21.

27. Saab, S., Alabbas, M., Samaha, R., Jaafar, R., Saab, SJr. Setting the boundaries of covid-19 lockdown relaxation measures. Library Hi Tech. (2021). Doi: 10.1108/LHT-03-2021-0095.

28. Deeb, O.E., Jalloul, M. The dynamics of COVID-19 spread: evidence from Lebanon. Math Biosci Eng.17(5):5618-5632. (2020). doi: 10.3934/mbe.2020302. PMID: 33120569

29. Tricco, A.C., Lillie, E., Zarin, W., et al. PRISMA Extension for Scoping Reviews (PRISMA-ScR): Checklist and Explanation. Ann Intern Med.169(7):467. (2018).

30. Guyatt, G., Oxman, A.D., Sultan, S., Brozek, J., Glasziou, P., Alonso-Coello, P., et al. GRADE guidelines:11. Making and overall of confidence in effect estimates for a single outcomeand for all outcomes. J clin epidemiol. 66(2):151-157. (2013). Doi:10.1016/j.jclinepi.2012.01.006.

31. Secinaro, S., Calandra, D., Secinaro, A., Muthurangu, V., Biancone, P. The role of artificial intelligence in healthcare: a structured literature review. BMC Med Inform Decis Mak. 21(1):125. (2021). doi: 10.1186/s12911-021-01488-9

32. Kumar, S., Choudhary, S., Jain, A., Singh, K., Ahmadian, A., Bajuri, M.Y. Brain tumor classification using deep neural network and transfer learning. Brain Topogr.36(3):305-318. (2023). doi: 10.1007/s10548-023-00953-033.

33. Ghanem, M., Ghaith, A.K., Zamanian, C., Bon-Nieves, A., Bhandarkar, A., Bydon, M., Quiñones-Hinojosa, A. Deep Learning Approaches for Glioblastoma Prognosis in Resource-Limited Settings: A Study Using Basic Patient Demographic, Clinical, and Surgical Inputs. World Neurosurg. 175:e1089-e1109. (2023). doi: 10.1016/j.wneu.2023.04.072.

34. Halabi, R., Dakroub, F., Haider, M.Z., Patel, S., Amhaz, N.A., Reslan, M.A., Eid, A.H., Mechref, Y., Darwiche, N., Kobeissy, F., Omeis, I., Shaito, A.A. Unveiling a Biomarker Signature of Meningioma: The Need for a Panel of Genomic, Epigenetic, Proteomic, and RNA Biomarkers to Advance Diagnosis and Prognosis. Cancers (Basel).15(22):5339. (2023). doi: 10.3390/cancers15225339

35. Ullah, M.S., Khan, M.A., Almujally, N.A., Alhaisoni, M., Akram, T., Shabaz, M. BrainNet: a fusion assisted novel optimal framework of residual blocks and stacked autoencoders for multimodal brain tumor classification. Sci Rep. 14(1):5895. (2024). doi: 10.1038/s41598-024-56657-3.

36. Gumaei, A., Sammouda, R., Al-Rakhami, M., AlSalman, H., El-Zaart, A. Feature selection with ensemble learning for prostate cancer diagnosis from microarray gene expression. Health Informatics J. 27(1):1460458221989402. (2021). doi: 10.1177/1460458221989402.

37.Zafar, M., Sharif, M.I., Kadry, S., Bukhari, S.A.C., Rauf, H.T. Skin Lesion Analysis and Cancer Detection Based on Machine/Deep Learning Techniques: A Comprehensive Survey. Life (Basel). 13(1):146. (2023). doi: 10.3390/life13010146.

38. Zaylaa, A.J., Kourtian, S. Advancing Breast Cancer Diagnosis through Breast Mass Images, Machine Learning, and Regression Models. Sensors (Basel). 24(7):2312. (2024). doi: 10.3390/s24072312

39. Magdy, O., Abd Elaziz, M., Elgarayhi, A., Ewees, A.A., Sallah, M. Bone metastasis detection method based on improving golden jackal optimization using whale optimization algorithm. Sci Rep. 13(1):15019. (2023). doi: 10.1038/s41598-023-41733-x

40. Dhasmana, A., Dhasmana, S., Agarwal, S., Khan, S., Haque, S., Jaggi, M., Yallapu, M.M., Chauhan, S.C. Integrative big transcriptomics data analysis implicates crucial role of MUC13 in

pancreatic cancer. Comput Struct Biotechnol J. 21:2845-2857. (2023). doi: 10.1016/j.csbj.2023.04.029.

41. Rajinikanth, V., Kadry, S., Mohan, R., Rama, A., Khan, M.A., Kim, J. Colon histology slide classification with deep-learning framework using individual and fused features. Math Biosci Eng. 20(11):19454-19467. (2023). doi: 10.3934/mbe.2023861

42. Yagin, B., Yagin, F.H., Colak, C., Inceoglu, F., Kadry, S., Kim, J. Cancer Metastasis Prediction and Genomic Biomarker Identification through Machine Learning and eXplainable Artificial Intelligence in Breast Cancer Research. Diagnostics (Basel). 13(21):3314. (2023). doi: 10.3390/diagnostics13213314.

43. Walsh, J.L., AlJaroudi, W.A., Lamaa, N., Abou Hassan, O.K., Jalkh, K., Elhajj, I.H., Sakr, G., Isma'eel, H. A speckle-tracking strain-based artificial neural network model to differentiate cardiomyopathy type. Scand Cardiovasc J. 54(2):92-99. (2020). doi: 10.1080/14017431.2019.1678764

44. Ahmad, A., Ibrahim, Z., Sakr, G., El-Bizri, A., Masri, L., Elhajj, I.H., El-Hachem, N., Isma'eel, H. A comparison of artificial intelligence-based algorithms for the identification of patients with depressed right ventricular function from 2-dimentional echocardiography parameters and clinical features. Cardiovasc Diagn Ther. 10(4):859-868. (2020). doi: 10.21037/cdt-20-471

45. Helwan, A., Azar, D., Ma'aitah, M.K.S. Conventional and deep learning methods in heart rate estimation from RGB face videos. Physiol Meas.45(2). (2024). doi: 10.1088/1361-6579/ad1458 46.

46. Guldogan, E., Yagin, F.H., Pinar, A., Colak, C., Kadry, S., Kim, J. A proposed tree-based explainable artificial intelligence approach for the prediction of angina pectoris. Sci Rep. 13(1):22189. (2023). doi: 10.1038/s41598-023-49673-2

47. Mitu, M., Hasan, S.M.M., Uddin, M.P., Mamun, M.A., Rajinikanth, V., Kadry, S. A stroke prediction framework using explainable ensemble learning. Comput Methods Biomech Biomed Engin.1-20. (2024). doi: 10.1080/10255842.2024.2316877

48. Li, B., Nassereldine, R., Zamzam, A., Syed, M.H., Mamdani, M., Al-Omran, M., Abdin, R., Qadura, M. Development and evaluation of a prediction model for peripheral artery disease-related major adverse limb events using novel biomarker data. J Vasc Surg. S0741-5214(24)00951-0. (2024). doi: 10.1016/j.jvs.2024.03.450

49. Helwan, A., Ma'aitah, M.K.S., Hamdan, H., Ozsahin, D.U., Tuncyurek, O. Radiologists versus Deep Convolutional Neural Networks: A Comparative Study for Diagnosing COVID-19. Comput Math Methods Med.:5527271. (2021). doi: 10.1155/2021/5527271

50. Rashid, S., Raza, M., Sharif, M., Azam, F., Kadry, S., Kim, J. White blood cell image analysis for infection detection based on virtual hexagonal trellis (VHT) by using deep learning. Sci Rep. 13(1):17827. (2023). doi: 10.1038/s41598-023-44352-8.

51. Tarek, Z., Shams, M.Y., Towfek, S.K., Alkahtani, H.K., Ibrahim, A, Abdelhamid AA, Eid MM, Khodadadi N, Abualigah L, Khafaga DS, Elshewey AM. An Optimized Model Based on Deep Learning and Gated Recurrent Unit for COVID-19 Death Prediction. Biomimetics (Basel). 8(7):552. (2023). doi: 10.3390/biomimetics8070552.

52. Amin, J., Anjum M.A., Ahmad, A., Sharif, M.I., Kadry, S., Kim, J. Microscopic parasite malaria classification using best feature selection based on generalized normal distribution optimization. PeerJ Comput Sci. 10:e1744. (2024). doi: 10.7717/peerj-cs.1744

53. Ngugi, H.N., Ezugwu, A.E., Akinyelu, A.A., Abualigah, L. Revolutionizing crop disease detection with computational deep learning: a comprehensive review. Environ Monit Assess. 196(3):302. (2024). doi: 10.1007/s10661-024-12454-z

54. Javed, A.R., Khan, H.U., Alomari, M.K.B., Sarwar, M.U., Asim, M., Almadhor, A.S., Khan, M.Z. Toward explainable AI-empowered cognitive health assessment. Front Public Health.11:1024195. (2023). doi: 10.3389/fpubh.2023.1024195

55. Jaber, D., Hajj, H., Maalouf, F., El-Hajj, W. Medically-oriented design for explainable AI for stress prediction from physiological measurements. BMC Med Inform Decis Mak. 22(1):38. (2022). doi: 10.1186/s12911-022-01772-2.

56. Ramzan, M., Raza, M., Sharif ,M.I., Azam, F., Kim, J., Kadry, S. Gastrointestinal tract disorders classification using ensemble of InceptionNet and proposed GITNet based deep feature with ant colony optimization. PLoS One.18(10):e0292601. (2023). doi: 10.1371/journal.pone.0292601

57. Ali, L., Javeed, A., Noor, A., Rauf, H.T., Kadry, S., Gandomi, A.H. Parkinson's disease detection based on features refinement through L1 regularized SVM and deep neural network. Sci Rep.14(1):1333. (2024). doi: 10.1038/s41598-024-51600-y

58. Voigtlaender, S., Pawelczyk, J., Geiger, M., Vaios, E.J., Karschnia, P., Cudkowicz, M., et al. Artificial intelligence in neurology: opportunities, challenges, and policy implications. J Neurol. 271(5):2258-2273. (2024). doi: 10.1007/s00415-024-12220-8

59. Hallal, M., Awad, M., Khoueiry, P. TempoMAGE: a deep learning framework that exploits the causal dependency between time-series data to predict histone marks in open chromatin regions at time-points with missing ChIP-seq datasets. Bioinformatics. 37(23):4336-4342. (2012). doi: 10.1093/bioinformatics/btab513

60. de Brevern, A.G., Rebehmed, J. Current status of PTMs structural databases: applications, limitations and prospects. Amino Acids.54(4):575-590. (2022). doi: 10.1007/s00726-021-03119-z

61. Hussain, M., Khan, M.A., Damaševičius, R., Alasiry, A., Marzougui, M., Alhaisoni, M., Masood, A. SkinNet-INIO: Multiclass Skin Lesion Localization and Classification Using Fusion-Assisted Deep Neural Networks and Improved Nature-Inspired Optimization Algorithm. Diagnostics (Basel).13(18):2869. (2023). doi: 10.3390/diagnostics13182869

62. Bibi, S., Khan, M.A., Shah, J.H., Damaševičius, R., Alasiry, A., Marzougui, M., Alhaisoni, M., Masood, A. MSRNet: Multiclass Skin Lesion Recognition Using Additional Residual Block Based Fine-Tuned Deep Models Information Fusion and Best Feature Selection. Diagnostics (Basel). 13(19):3063. (2023). doi: 10.3390/diagnostics13193063

63. Al-Sheikh, M.H., Al Dandan, O., Al-Shamayleh, A.S., Jalab, H.A., Ibrahim, R.W. Multi-class deep learning architecture for classifying lung diseases from chest X-Ray and CT images. Sci Rep.13(1):19373. (2023). doi: 10.1038/s41598-023-46147-3

64. Askin, S., Burkhalter, D., Calado, G., El Dakrouni, S. Artificial Intelligence Applied to clinical trials: opportunities and challenges. Health Technol (Berl).13(2):203-213. (2023). doi: 10.1007/s12553-023-00738-2

65. Venkatapathappa, P., Sultana, A., K, S.V., Mansour, R., Chikkanarayanappa, V., Rangareddy, H. Ocular Pathology and Genetics: Transformative Role of Artificial Intelligence (AI) in Anterior Segment Diseases. Cureus.16(2):e55216. (2024). doi: 10.7759/cureus.55216.

66. Barakett-Hamade, V., Ghayad, J.P., Mchantaf, G., Sleilaty, G. Is Machine Learning-derived Low-Density Lipoprotein Cholesterol estimation more reliable than standard closed form equations? Insights from a laboratory database by comparison with a direct homogeneous assay. Clin Chim Acta.519:220-226. (2021). doi: 10.1016/j.cca.2021.05.008.

67. Li, B., Verma, R., Beaton, D., Tamim, H., Hussain, M.A., Hoballah, J.J., Lee, D.S., Wijeysundera, D.N., de Mestral, C., Mamdani, M., Al-Omran, M. Predicting outcomes following open revascularization for aortoiliac occlusive disease using machine learning. J Vasc Surg. 78(6):1449-1460.e7. (2023). doi: 10.1016/j.jvs.2023.07.006

68. Li, B., Verma, R., Beaton, D., Tamim, H., Hussain, M.A., Hoballah, J.J., et al. Predicting Major Adverse Cardiovascular Events Following Carotid Endarterectomy Using Machine Learning. J Am Heart Assoc.12(20):e030508. (2023). doi: 10.1161/JAHA.123.030508

69. Li, B., Aljabri, B., Verma, R., Beaton, D., Eisenberg, N., Lee, D.S., et al. Machine learning to predict outcomes following endovascular abdominal aortic aneurysm repair. Br J Surg.110(12):1840-1849. (2023). doi: 10.1093/bjs/znad287

70. Li, B., Verma, R., Beaton, D., Tamim, H., Hussain, M.A., Hoballah, J.J., et al. Predicting outcomes following lower extremity open revascularization using machine learning. Sci Rep.14(1):2899. (2024). doi: 10.1038/s41598-024-52944-1.

71. Hammoud, B., El Zini, J., Awad, M., Sweid, A., Tjoumakaris, S., Jabbour, P. Predicting incomplete occlusion of intracranial aneurysms treated with flow diverters using machine learning models. J Neurosurg.140(6):1716-1725. (2023). doi: 10.3171/2023.9.JNS231031. PMID: 38039540.

72. Qasrawi, R., Amro, M., VicunaPolo, S., Abu Al-Halawa, D., Agha, H., Abu Seir, R., et al. Machine learning techniques for predicting depression and anxiety in pregnant and postpartum women during the COVID-19 pandemic: a cross-sectional regional study. F1000Res.11:390. (2022). doi: 10.12688/f1000research.110090.1.

73. Mahalingam, M., Jammal, M., Hoteit, R., Ayna, D., Romani, M., Hijazi, S., Bou-Hamad, I., El Morr, C. A Machine Learning Study to Predict Anxiety on Campuses in Lebanon. Stud Health Technol Inform.305:85-88. (2023). doi: 10.3233/SHTI230430

74. El Morr, C., Jammal, M., Bou-Hamad, I., Hijazi, S., Ayna, D., Romani, M., Hoteit, R. Predictive Machine Learning Models for Assessing Lebanese University Students' Depression, Anxiety, and Stress During COVID-19. J Prim Care Community Health.15:21501319241235588. (2024). doi: 10.1177/21501319241235588.

75. Rammal, A., Assaf, R., Goupil, A., Kacim, M., Vrabie, V. Machine learning techniques on homological persistence features for prostate cancer diagnosis. BMC Bioinformatics.23(1):476. (2022). doi: 10.1186/s12859-022-04992-5

76. Ghait,h A.K., Ghanem, M., Zamanian, C., Bon-Nieves, A.A., Bhandarkar, A., Nathani, K., Bydon, M., Quiñones-Hinojosa, A. Using machine learning to predict 30-day readmission and reoperation following resection of supratentorial high-grade gliomas: an ACS NSQIP study involving 9418 patients. Neurosurg Focus. 54(6):E12. (2023). doi: 10.3171/2023.3.FOCUS22652

77. Ghazi, L., Farhat, K., Hoenig, M.P., Durant, T.J.S, El-Khoury, J.M. Biomarkers vs Machines: The Race to Predict Acute Kidney Injury. Clin Chem.70(6):805-819. (2024). doi: 10.1093/clinchem/hvad217. PMID: 38299927.

78. Ghanem, M., Ghaith, A.K., El-Hajj, V.G., Bhandarkar, A., de Giorgio, A., Elmi-Terander, A., Bydon, M. Limitations in Evaluating Machine Learning Models for Imbalanced Binary Outcome Classification in Spine Surgery: A Systematic Review. Brain Sci. 13(12):1723. (2023). doi: 10.3390/brainsci13121723

79. Hammoud, B., Semaan, A., Elhajj, I., Benova, L. Can machine learning models predict maternal and newborn healthcare providers' perception of safety during the COVID-19 pandemic? A cross-sectional study of a global online survey. Hum Resour Health.20(1):63. (2022). doi: 10.1186/s12960-022-00758-5

80. Chedid, N., Tabbal, J., Kabbara, A., Allouch, S., Hassan, M. The development of an automated machine learning pipeline for the detection of Alzheimer's Disease. Sci Rep.12(1):18137. (2022). doi: 10.1038/s41598-022-22979-3

81. Saab, A., Abi Khalil, C., Jammal, M., Saikali, M., Lamy, J.B. Early Prediction of All-Cause Clinical Deterioration in General Wards Patients: Development and Validation of a Biomarker-Based Machine Learning Model Derived From Rapid Response Team Activations. J Patient Saf.18(6):578-586. (2022). doi: 10.1097/PTS.0000000000001069.

82. Saab, A., Saikali, M., Lamy, J.B. Comparison of Machine Learning Algorithms for Classifying Adverse-Event Related 30-Day Hospital Readmissions: Potential Implications for Patient Safety. Stud Health Technol Inform.272:51-54. (2020). doi: 10.3233/SHTI200491

83. Felefly, T., Roukoz, C., Fares, G., Achkar, S., Yazbeck, S., Meyer, P., Kordahi, M., Azoury, F., Nasr, D.N., Nasr, E., Noël, G., Francis, Z. An Explainable MRI-Radiomic Quantum Neural Network to Differentiate Between Large Brain Metastases and High-Grade Glioma Using Quantum Annealing for Feature Selection. J Digit Imaging. 36(6):2335-2346. (2023). doi: 10.1007/s10278-023-00886-x.

84. Lucchetti, D., Colella, F., Artemi, G., Haque, S., Sgambato, A., Pellicano, R., Fagoonee, S. Smart nano-sized extracellular vesicles for cancer therapy: Potential theranostic applications in gastrointestinal tumors. Crit Rev Oncol Hematol.191:104121. (2023). doi: 10.1016/j.critrevonc.2023.104121

85. Hage Chehade, A., Abdallah, N., Marion, J.M., Oueidat, M., Chauvet, P. Lung and colon cancer classification using medical imaging: a feature engineering approach. Phys Eng Sci Med. 45(3):729-746. (2022). doi: 10.1007/s13246-022-01139-x

86. Dangi, P., Chaudhary, N., Chaudhary, V., Virdi, A.S., Kajla, P., Khanna, P., et al. Nanotechnology impacting probiotics and prebiotics: a paradigm shift in nutraceuticals technology. Int J Food Microbiol.388:110083. (2023). doi: 10.1016/j.ijfoodmicro.2022.110083.

87. Dasegowda, G., Kalra, M.K., Abi-Ghanem, A.S., Arru, C.D., Bernardo, M., et al. Suboptimal Chest Radiography and Artificial Intelligence: The Problem and the Solution. Diagnostics (Basel).13(3):412. (2023). doi: 10.3390/diagnostics13030412.

88. Acharya, V., Dhiman, G., Prakasha, K., Bahadur, P., Choraria, A., M, S., et al. AI-Assisted Tuberculosis Detection and Classification from Chest X-Rays Using a Deep Learning Normalization-Free Network Model. Comput Intell Neurosci.2022:2399428. (2022). doi: 10.1155/2022/2399428.

89. Alnazer, I., Bourdon, P., Urruty, T., Falou, O., Khalil, M., Shahin, A., et al. Recent advances in medical image processing for the evaluation of chronic kidney disease. Med Image Anal.69:101960. (2021). doi: 10.1016/j.media.2021.101960

90. Serhal, H., Abdallah, N., Marion, J.M., Chauvet, P., Oueidat, M., Humeau-Heurtier, A. Overview on prediction, detection, and classification of atrial fibrillation using wavelets and AI on ECG. Comput Biol Med.142:105168. (2022). doi: 10.1016/j.compbiomed.2021.105168

91. Moshawrab, M., Adda, M., Bouzouane, A., Ibrahim, H., Raad, A. Smart Wearables for the Detection of Cardiovascular Diseases: A Systematic Literature Review. Sensors (Basel). 23(2):828. (2023). doi: 10.3390/s23020828

92. Boulos, L. J., Mendes, A., Delmas, A., Chraibi Kaadoud, I. An Iterative and Collaborative End-to-End Methodology Applied to Digital Mental Health. Frontiers in psychiatry, 12, 574440. (2021) https://doi.org/10.3389/fpsyt.2021.574440

93. Kabbara, A., Robert, G., Khalil, M., Verin, M., Benquet, P., Hassan, M. An electroencephalography connectome predictive model of major depressive disorder severity. Sci Rep. 2022;12(1):6816. doi: 10.1038/s41598-022-10949-8

94. Malik, S., Waheed, Y. Emerging Applications of Nanotechnology in Dentistry. Dent J (Basel). 11(11):266. (2023). doi: 10.3390/dj11110266

95. Jerbaka, M., Laganà, A.S., Petousis, S., Mjaess, G., Ayed, A., Ghezzi, F., Terzic, S., Sleiman, Z. Outcomes of robotic and laparoscopic surgery for benign gynaecological disease: a systematic review. J Obstet Gynaecol. 42(6):1635-1641. (2022). doi: 10.1080/01443615.2022.2070732

96. Yammine, K., Karbala, J., Maalouf, A., Daher, J., Assi, C. Clinical outcomes of the use of 3D printing models in fracture management: a meta-analysis of randomized studies. Eur J Trauma Emerg Surg.48(5):3479-3491. (2022). doi: 10.1007/s00068-021-01758-1

97. Atat, O.E., Farzaneh, Z., Pourhamzeh, M., Taki, F., Abi-Habib, R., Vosough, M., El-Sibai, M. 3D modeling in cancer studies. Hum Cell. 35(1):23-36. (2022). doi: 10.1007/s13577-021-00642-9.

98. Saleh, F., Harb, A., Soudani, N., Zaraket, H. A three-dimensional A549 cell culture model to study respiratory syncytial virus infections. J Infect Public Health.13(8):1142-1147. (2020). doi: 10.1016/j.jiph.2020.03.011

99. Malik, S., Muhammad, K., Waheed, Y. Emerging Applications of Nanotechnology in Healthcare and Medicine. Molecules. 28(18):6624. (2023). doi: 10.3390/molecules28186624

100. R,W., Malik, T., Khoei, A.A., Salihu, H.M. Artificial Intelligence in Global Health -A Framework and Strategy for Adoption and Sustainability. Int J MCH AIDS.9(1):121-127. (2020). doi: 10.21106/ijma.296

101. Har,a N., Solomon, P., Kim, S.L., Sonnenwald, A. An emerging view of scientific collaboration: scientists' perspectives on collaboration and factors that impact collaboration. J Am Soc Inf Sci Tech.54(10):952–965. (2003). doi: 10.1002/asi.10291

102. Ramu, S.P., Srivastava, G., Chengoden, R., Victor, N., Maddikunta, P., Gadekallu, T. The metaverse for cognitive health: a paradigm shift. IEEE Consumer Electronics Magazine.13(3):73-79. (2024). doi: 10.1109/MCE.2023.3289034

103. Chengoden, R., Victor, N., Huynh-The, T., Yenduri, G., Jhaveri, R., Alazab, M. et al. Metaverse for Healthcare: A Survey on Potential Applications, Challenges and Future Directions. IEEE Access. 11: 12765 12795. (2023). https://doi.org/10.1109/ACCESS.2023.3241628

104. Zamzami, I., Pathoee, K., Gupta, B., Mishra, A., Rawat, D., Alhalabi, W., Machine learning algorithms for smart and intelligent healthcare system in Society 5.0. Int J Int Sys. 37. (2022). Doi:10.1002/int.23061.

105. Javaid, M., Haleem, H., Singh, R.P., Suman, R., Rab, S. Significance of machine learning in healthcare: Features, pillars and applications, Int J Int Net. 3:58-73. (2022). https://doi.org/10.1016/j.ijin.2022.05.002.

106. Habehh, H., Gohel, S. Machine Learning in Healthcare. Current genomics.22(4):291–300. (2021). https://doi.org/10.2174/1389202922666210705124359

107. Liu, C., Li, Y., Fang, M., Liu, F. Using machine learning to explore the determinants of service satisfaction with online healthcare platforms during the COVID-19 pandemic. Service Business. 17(2):449–476. (2023). https://doi.org/10.1007/s11628-023-00535-x

108. Sahu, H., Kashyap, R., Bhatia, S., Dewangan, B.K., Alkhaldi, N.A., Anosh Babu, S., Mohan, S. Analysis of deep learning methods for healthcare sector - medical imaging disease detection. Contemp. Math. 4(4):830-852. (2023). https://ojs.wiserpub.com/index.php/CM/article/view/2496

109. Biancone, P., Secinaro, S., Brescia, V., Calandra, D. Management of open innovation in healthcare for cost accounting using EHR. J Open Innov Technol Mark Complex.5:99. (2019). https://doi.org/10.3390/joitmc5040099

110.Gupta, B., Gaurav, A., Panigrahi, P.K., Arya, V. Analysis of cutting-edge technologies for enterprise information system and management. Enterprise Inf Syst. 17(11). (2023). https://doi.org/10.1080/17517575.2023.2197406

111. Letourneau-Guillon, L., Camirand, D., Guilbert, F., Forghani, R. Artificial Intelligence Applications for Workflow, Process Optimization and Predictive Analytics. Neuroimaging Clin N Am.30(4):e1-e15. (2020). doi: 10.1016/j.nic.2020.08.008

112. Sarraf, S., Desouza, D.D., Anderson, J., Saverino, C. Alzheimer's Disease Neuroimaging Initiative. MCADNNet: Recognizing Stages of Cognitive Impairment through Efficient Convolutional fMRI and MRI Neural Network Topology Models. IEEE Access.7:155584-155600. (2019). doi: 10.1109/ACCESS.2019.2949577

113. Chakradhar, S. Predictable response: Finding optimal drugs and doses using artificial intelligence. Nat Med. 23(11):1244-1247. (2017). doi: 10.1038/nm1117-1244.

114. Khan, Y.F., Kaushik, B., Chowdhary, C.L., Srivastava, G. Ensemble Model for Diagnostic Classification of Alzheimer's Disease Based on Brain Anatomical Magnetic Resonance Imaging. Diagnostics (Basel). 12(12):3193. (2022). doi: 10.3390/diagnostics12123193
115. Agrawal, A., Gans, J.S., Goldfarb, A. Exploring the impact of artificial intelligence: prediction versus judgment. Inf Econ Policy.1(47):1–6. (2019). https://doi.org/10.1016/j.infoecopol.2019.05.001.
116. Siddiqui, M.F., Alam, A., Kalmatov, R., Mouna, A., Villela, R., Mitalipova, A. Leveraging healthcare system with nature-inspired computing techniques: an overview and future perspective. Studies in Computational Intelligence. 1066. (2023). https://doi.org/10.1007/978-981-19-6379-7_2
117. Kaur, A., Garg, R., Gupta, P. Challenges facing AI and Big data for Resource-poor Healthcare System. 2021 Second International Conference on Electronics and Sustainable Communication Systems (ICESC).1426-1433. (2021). doi: 10.1109/ICESC51422.2021.9532955.
118. Khan, B., Fatima, H., Qureshi, A., Kumar, S., Hanan, A., Hussain, J., Abdullah, S. Drawbacks of Artificial Intelligence and Their Potential Solutions in the Healthcare Sector. Biomed Mater Devices.: 1-8. (2023). doi: 10.1007/s44174-023-00063-2
119. Madanan, M., Zulkefli, N., Velayudhan, N. Designing a Hybrid Artificial Intelligent Clinical Decision Support System Using Artificial Neural Network and Artificial Bee Colony for Predicting Heart Failure Rate. 2021 International Conference on Computer Communication and Informatics (ICCCI):1-7. (2021). doi: 10.1109/ICCCI50826.2021.9457007.
120. Srivastava, B., Rossi, F. Rating AI systems for bias to promote trustable applications. IBM J Res Develop.63(4/5):5:1-5. (2019). doi: 10.1147/JRD.2019.2935966
121. Hazime, A., Fakhoury, M. Chapter 18 - Artificial intelligence in psychiatry: current practice and major challenges, Editor(s): Chayakrit Krittanawong, Artificial Intelligence in Clinical Practice, Academic Press:163-167, ISBN 9780443156885. (2024). https://doi.org/10.1016/B978-0-443-15688-5.00015-2.
122. Sallam, M. ChatGPT Utility in Healthcare Education, Research, and Practice: Systematic Review on the Promising Perspectives and Valid Concerns. Healthcare (Basel). 11(6):887. (2023). doi: 10.3390/healthcare11060887.
123. Bashir, A.K., Victor, N., Bhattacharya, S., Huynh-The, T., Chengoden, R., Yenduri, G. Federated Learning for the Healthcare Metaverse: concepts, applications, challenges, and future

directions. IEEE Internet of Things J. 10(24):21873-21891. (2023). doi: 10.1109/JIOT.2023.3304790.

124. Halwani, J., Mouawad D. Implementation of e-health innovative technologies in North Lebanon hospitals. East Med Health J. 27(9), 892–898. (2021). https://doi.org/10.26719/emhj.21.030

Figure

Click here to access/download;Figure;Figure 1.tif

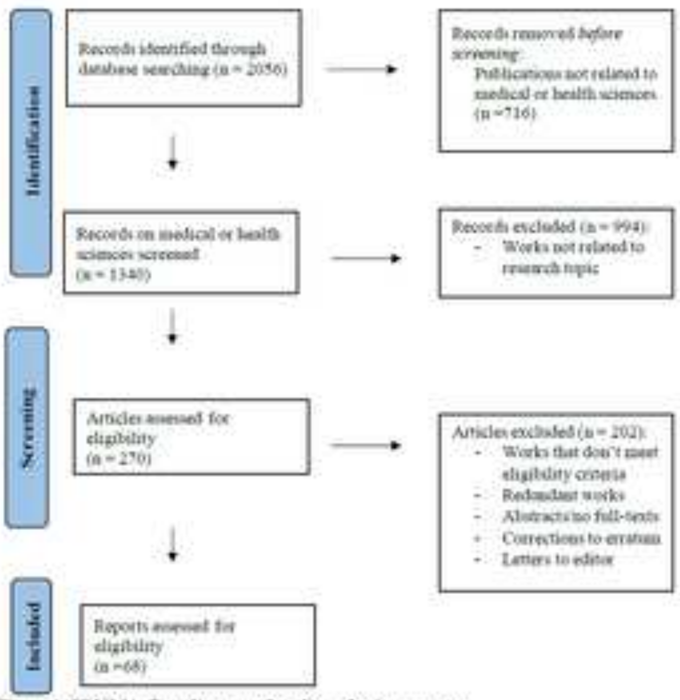

Figure 1. PRISMA flow diagram of studies selection process

Figure Click here to access/download;Figure;Figure 2.tif

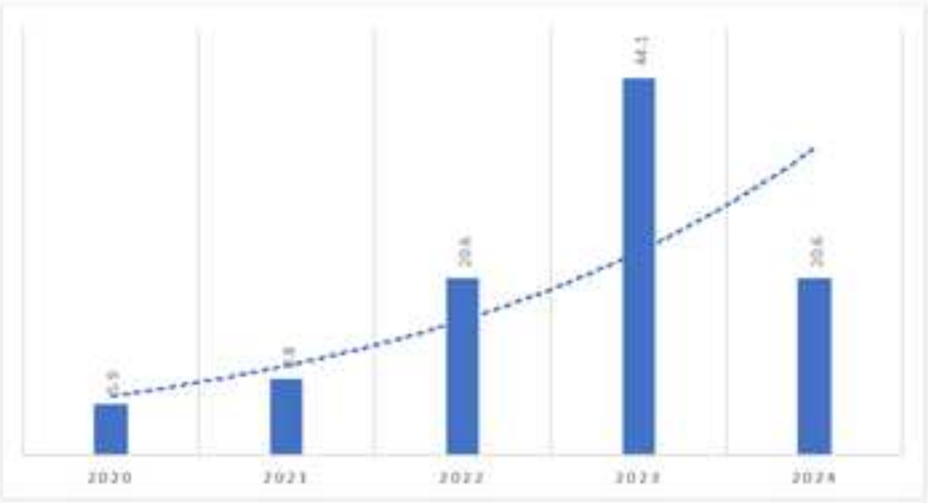

Figure 2. Percentage of AI in health studies across the years

Figure

Click here to access/download;Figure;Figure 3.tif

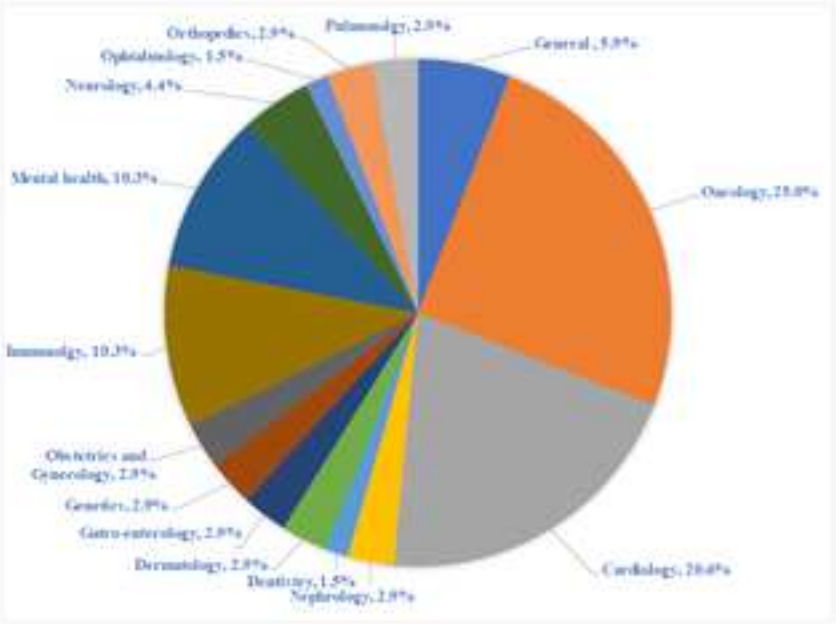

Figure 3. Main areas of AI application

Figure

Click here to access/download;Figure;Figure 4.tif

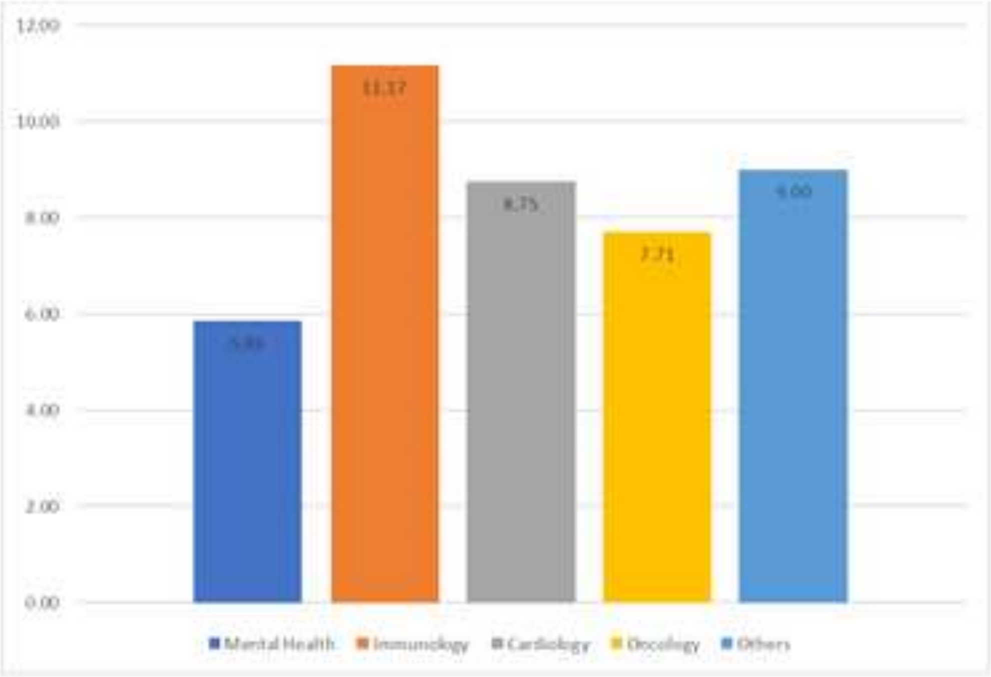

Figure 4. Differences in the average number of citations among different medical fields

Click here to access/download
Supporting Information
Prisma Checklist.docx
