# Supplementary material for: Investigating the Key Trends in Applying Artificial Intelligence to Health Technologies: A Scoping Review
Source: PLoS One. 2025 May 15;20(5):e0322197. doi: 10.1371/journal.pone.0322197 (PMC12080793; doi:10.1371/journal.pone.0322197)
Supplement: Attachment — Submitted filename: Reviewers comments -AI in Healthcare-.pdf [file pone.0322197.s002.pdf]

Congratulations to the authors for completing there research and submitting it for publication.

Kindly refrain from mimicking the suggested changes as it is.

Overall, I recommend that the article should be copy-edited by a professional to address minor grammatical, punctuation and typo mistakes.

Abstract:
- The aims and objectives should be defined more clearly. For instance, rather than stating, “This study presents a scoping review of …,” the authors should elaborate on the necessity of this study.

Introduction:
The introduction is well-structured and provides a clear research question and objectives. However, some refinements are suggested:

Line 63: In the sentence, “Certain devices, utilizing an interdisciplinary approach …,” the authors should provide examples or names of these devices and include appropriate citations.

Line 73: The sentence, “AI has become an indispensable tool in various medical applications, revolutionizing traditional practices,” should be removed as it reiterates content already covered in previous sentences.

Line 74: The sentence, “Several studies exemplify the integration of artificial intelligence into medicine,” appears overstated and adds little value. Consider removing it.

Line 75: Sentence "artificially intelligent computer systems inpatient diagnosis" likely contains a typo. It should read "artificially intelligent computer systems in patient diagnosis."

Line 87: The sentence, “The Middle East and North Africa region acknowledged the vital importance of adapting…..” is quite lengthy. For improved readability, it can be split into two sentences. For example: “The Middle East and North Africa Region acknowledged the vital importance of adapting to new technologies. They recognized the key role of these technologies in transforming the region and advancing to the forefront of the digital economy and healthcare.”

Line 97: Discussion of Lebanon’s setbacks during COVID-19 can be made more concise by removing jargon.

Line 110: There is a minor grammatical error,
it should be “What AI methodologies have been applied for healthcare systems in Lebanon?"

Materials and Methods:
Line 117-118: Ensure a consistent format for the year range filter, such as "2020 to 2024" or "January 2020 to April 2024."

- In the section “Publication Characteristics,” it is mentioned that studies were selected from 2020 to 2023. For clarity, use a clear and consistent range for reviewers and readers.

Line 118: Replace “following keywords” with “these keywords” for technical accuracy.

Lines 127-130: The sentence discussing the choice of scoping review over systematic review could be rephrased to present a more positive perspective. For instance: "A scoping review was chosen over a systematic review because it allows for a broader exploration of the literature and identification of key concepts and research gaps, which is more suitable for this study given the heterogeneity of the included studies."

Line 149: Replace the typo "v7" with "(7)."

Line 167: The sentence, “Main findings from the studies were reviewed using descriptive and analytical methods based on different variables outcomes,” should specify the variables for clarity.

Results:
Line 187: The sentence, “The most common reasons for excluding some journal articles after full-text review …,” could be made more specific.
For example: "The most common reasons for excluding articles after full-text review included lack of relevance to the research question and AI not being the primary focus or methodology."

Line 231: There is a discrepancy between the number of articles per quartile mentioned in the text and Table 2. This should be reconciled.

Line 244: Replace “didn't” with “did not” to align with academic writing standards.

Lines 253-255: Clarify how many studies fall under each domain to ensure better understanding for the general audience.

Discussion:
Lines 376-382: Concise this paragraph to 2-3 sentences.

Lines 388-391: This paragraph could benefit from restructuring for smoother flow.
For example: "Our findings indicate that AI applications in healthcare are predominantly focused on diagnosing and predicting diseases such as cancer, cardiovascular diseases, infectious diseases, and neurological disorders. This focus enhances patient care by allowing healthcare professionals to spend more time with patients, adopt a holistic care approach, and improve patient satisfaction."

Lines 422-423: Strengthen the sentence by briefly summarizing the specific challenges reported in the cited studies.

Conclusion:
Line 475: Specify that the study focuses on Lebanon rather than referring to an unspecified Middle Eastern country.
